# Effect of N-Acetylcysteine on Sleep: Impacts of Sex and Time of Day

**DOI:** 10.3390/antiox12051124

**Published:** 2023-05-19

**Authors:** Priyanka N. Bushana, Michelle A. Schmidt, Kevin M. Chang, Trisha Vuong, Barbara A. Sorg, Jonathan P. Wisor

**Affiliations:** 1 Elson S. Floyd College of Medicine, Washington State University, Spokane, WA 99202, USA; priyanka.bushana@wsu.edu (P.N.B.); maschmidt@wsu.edu (M.A.S.); kevin.m.chang@wsu.edu (K.M.C.); trisha.vuong@wsu.edu (T.V.); 2 R.S. Dow Neurobiology Laboratories, Legacy Research Institute, Portland, OR 97232, USA; bsorg@downeurobiology.org

**Keywords:** electroencephalography, sleep, antioxidants, schizophrenia, N-acetylcysteine

## Abstract

Non-rapid eye movement sleep (NREMS) is accompanied by a decrease in cerebral metabolism, which reduces the consumption of glucose as a fuel source and decreases the overall accumulation of oxidative stress in neural and peripheral tissues. Enabling this metabolic shift towards a reductive redox environment may be a central function of sleep. Therefore, biochemical manipulations that potentiate cellular antioxidant pathways may facilitate this function of sleep. N-acetylcysteine increases cellular antioxidant capacity by serving as a precursor to glutathione. In mice, we observed that intraperitoneal administration of N-acetylcysteine at a time of day when sleep drive is naturally high accelerated the onset of sleep and reduced NREMS delta power. Additionally, N-acetylcysteine administration suppressed slow and beta electroencephalographic (EEG) activities during quiet wake, further demonstrating the fatigue-inducing properties of antioxidants and the impact of redox balance on cortical circuit properties related to sleep drive. These results implicate redox reactions in the homeostatic dynamics of cortical network events across sleep/wake cycles, illustrating the value of timing antioxidant administration relative to sleep/wake cycles. A systematic review of the relevant literature, summarized herein, indicates that this “chronotherapeutic hypothesis” is unaddressed within the clinical literature on antioxidant therapy for brain disorders such as schizophrenia. We, therefore, advocate for studies that systematically address the relationship between the time of day at which an antioxidant therapy is administered relative to sleep/wake cycles and the therapeutic benefit of that antioxidant treatment in brain disorders.

## 1. Introduction

Sleep propensity and its electroencephalographic manifestations increase as a function of time spent awake and decline as a function of time spent asleep. The accumulation of sleep needs during wakefulness and its discharge in sleep is thus said to be a homeostatic process [1]. The biochemical nature of this homeostatic process is not known. The homeostat may relate to the fundamental shift in cerebral metabolism away from the consumption of glucose to other energy sources. Glucose utilization requires a series of oxidation/reduction (redox) reactions, and it is thus conceivable that the homeostat is embodied, at least in part, by cellular redox status in the brain. The brain accumulates oxidative stress as a consequence of wakefulness, and this oxidative stress is reversed by sleep [2,3,4,5]. If brain redox status does indeed contribute to sleep homeostasis, then perturbation of brain redox status should alter the sleep homeostatic process. The goal of the current study was to measure the effects of systemic manipulations of redox substrates on EEG readout of sleep homeostasis.

As an electron carrier, nicotinamide adenine dinucleotide in its unphosphorylated (NAD) or phosphorylated (NAD phosphate; NADP) forms, is necessary for glycolytic metabolism and numerous other cellular biochemical pathways that rely on the transfer of electrons via redox reactions. NAD-dependent reactions occur at a high rate during wakefulness, as evidenced by the accumulation of NADH in brain tissues during sleep deprivation [6]. Parallel increases in markers of oxidative stress during sleep deprivation demonstrate that the capacity to undergo redox reactions is biochemically constrained during time spent awake by the availability of NAD+ as an oxidizing substrate [7,8].

**Figure 1:**
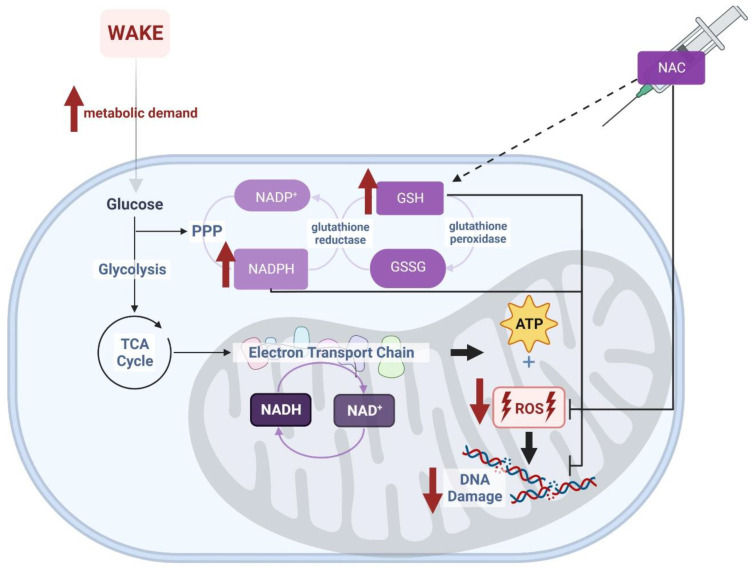
NAD(P)+/H are cofactors for hundreds of redox reactions involved in cellular metabolism. Exogenous application of NAC increases the pool of glutathione available in the cell for NADP(H)-dependent redox reactions. In turn, increased glutathione should produce additional opportunities for production of NADPH via the hexose monophosphate shunt [9]. Wakefulness challenges this system by promoting the accumulation of oxidative stress (i.e., reactive oxygen species [ROS] and DNA damage) during cellular metabolism. We propose that the increased availability of NADPH and glutathione as a result of NAC supplementation directly neutralizes the oxidative burden of wakefulness. Image created with BioRender.com.

Several strategies are available to increase the availability of NAD and related molecules as oxidizing substrates. The current study employed systemic administration of N-acetylcysteine (NAC), which directly increases the capacity for glutathione synthesis (Figure 1) to achieve this goal. NAC crosses the blood–brain barrier and, therefore, can undergo conversion to glutathione within cells of the brain parenchyma. We hypothesized that intraperitoneal (ip) injection of NAC via increased antioxidant capacity (i.e., glutathione) would decrease oxidative stress and subsequently decrease sleep needs. If, indeed, oxidative stress contributes to sleep homeostasis, this manipulation would be expected to manifest as an alteration in the homeostatic regulation of sleep. We tested this hypothesis by measuring sleep state timing and EEG parameters in animals subjected to NAC administration.

## 2. Materials and Methods

### 2.1. Ethical Approval

This study was approved by the Institutional Animal Care and Use Committee of Washington State University and conducted in accordance with National Institutes of Health’s Guidelines for the Care and Use of Laboratory Animals. All efforts were made to minimize the number of animals used in the experiments and to reduce the amount of pain and suffering.

### 2.2. Animals and Surgery

**Figure 2:**
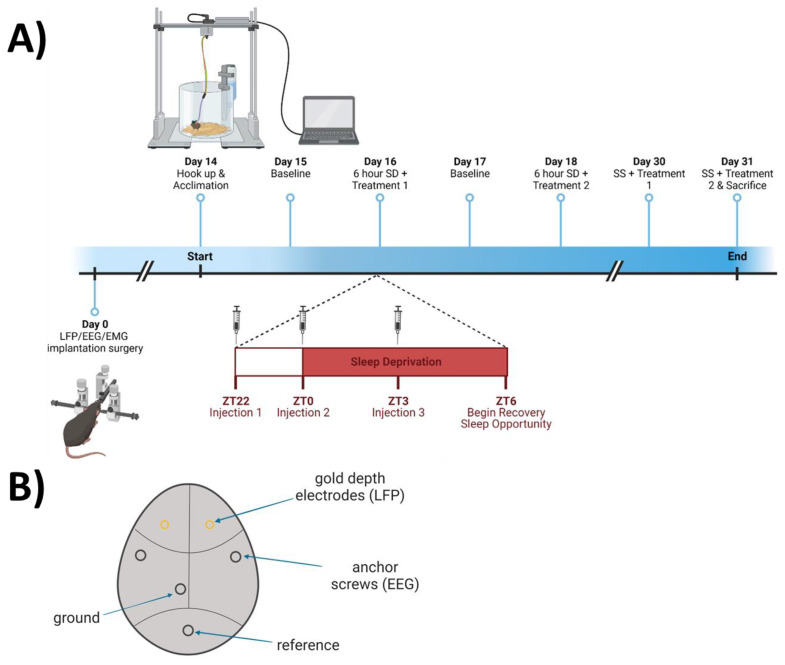
Experimental protocol for N-acetylcysteine (NAC) manipulation, as described in detail in the Section 2.3. (**A**) Schematic representation of experimental setting and timeline for experimentation. (**B**) Schematic representation of electrode placement. Image created with BioRender.com.

Twelve adult C57BL/6J mice (*n* = 12, 6 females, 6 males; all aged 10–12 weeks) were anesthetized using isoflurane (5% induction; 1–3% to maintain 0.5–1 Hz respiration rate) and placed in a stereotaxic frame. A 1-cm midline incision was made in the skin over the dorsal surface of the skull, and the skull was exposed to allow two holes, roughly 0.5 mm in diameter, to be drilled over predetermined coordinates targeting the medial prefrontal cortex (mPFC; A/P + 1.94; M/L ± 0.5; D/V − 1.3). At this location, stainless-steel polyimide-insulated depth electrodes (Plastics One part #E363/1/SPC diameter: 0.25 mm) were implanted bilaterally for local field potential (LFP) measurements. Prior to surgery, depth electrodes were cut to 1.5 mm. Mice were additionally implanted with EEG and electromyographic (EMG) electrodes as described previously [10] and diagrammed in Figure 2B. Briefly, two stainless-steel EEG screw electrodes were implanted over the parietal cortices, and two EMG electrodes were implanted in the nuchal muscles. All electrodes were soldered to a six-pin head mount connector and secured to the skull with dental cement. Buprenorphine SR (1.0 mg/kg) was administered once as a post-operative analgesic. After surgery, all mice were singly housed in a vivarium which remained between 70° and 75°F at a relative humidity of 50%, on an LD 12:12 cycle for 12–13 days during recovery from surgical procedures.

### 2.3. Experimental Design

Animals were subjected to saline (vehicle) and NAC treatments in a within-subjects crossover design, as described in Figure 2A. Two weeks after surgery, mice were connected to recording cables via a head mount and individually re-housed in cylindrical acrylic plastic cages 25 cm in diameter and 20 cm tall. Mice were allowed to habituate to the environment and head mount tethers overnight. Mice then underwent 24 h of undisturbed baseline LFP/EEG and EMG recording, starting 2 h before light onset (ZT22). After the baseline, three treatments of either NAC (600 mg/kg) or saline (12 mL/kg) intraperitoneal (i.p.) injections were applied (randomly assigned as Treatment 1 or Treatment 2) at ZT22, ZT0, and ZT3 (Figure 2A). Mice were concurrently sleep deprived (SD) for 6 continuous hours, from ZT0 to ZT6. Recovery sleep was recorded for the following 16 h before initiating a second session with the other treatment (at ZT 22, 0, and 3) and SD from ZT0-6. Two weeks later, mice were subjected to both treatments on the same time schedule as during the SD experiments but were allowed to sleep undisturbed between the injections (SS; spontaneous sleep). Each animal received both treatments concurrent with the two SD protocols in a repeated-measures, counterbalanced design (day 16 and 18; *n* = 12) and concurrent with the two SS protocols in a repeated-measures, counterbalanced design (day 30 and 31; *n* = 10) then was euthanized after the second spontaneous sleep recording (schematized in Figure 2A).

NAC is known to reach peak plasma levels quickly [11], with a half-life reported to range between 11 min and 6 h, depending on the animal and route of administration [12,13]. When administered intraperitoneally, NAC is directly taken up by the hepatic portal system and undergoes extensive first-pass metabolism through the liver before reaching main circulation and entering the brain [14]. The high dose used here (600 mg/kg) and the multiple injections delivered before and throughout sleep deprivation ensured that NAC was present systemically throughout the SD period. NAC was prepared in phosphate-buffered saline the day prior, balanced for pH (using NaOH), stored under nitrogen, and opened immediately prior to each injection to minimize oxidation.

### 2.4. Data Collection and Processing

Data from LFP, EEG, and EMG potentials were collected, extracted, and processed as described previously [10]. Briefly, LFP, EEG, and EMG signals from the head mount were fed through a PCB-based preamplifier (Part #8406-SL, Pinnacle Technology, Inc., Lawrence, KS, USA) to a commutator (Part #8408, Pinnacle Technology, Inc.), which was read into a PC-based acquisition system (Pinnacle Technology, Inc.; Part #8401). Signals were further amplified 50-fold and sampled at 400 Hz. LFP and EMG potentials were extracted using Sirenia software, version 2.2 from Pinnacle Technology, Inc. Sleep recording files were extracted in European Data format (.edf) and contained data from two frontal LFPs and one nuchal EMG.

Sleep recording files were scored through the online computational tool SPINDLE (sleep phase identification with neural networks for domain-invariant learning) [15]. SPINDLE allows .edf files to be uploaded to a web-based platform and processes signals automatically with a 4-s epoch resolution. The algorithm classifies each epoch as one of three vigilance states: wakefulness, non-rapid eye movement sleep (NREM sleep; NREMS; or slow wave sleep; SWS, which is assumed to be synonymous with NREMS in this manuscript), or rapid-eye-movement sleep (REM sleep; REMS) and additionally designates each epoch as likely or unlikely to contain artifacts. Briefly, the SPINDLE algorithm processes raw signals by windowed Fourier transforms, amongst other pre-processing methods, then feeds these data through a convolutional neural network to detect sleep states. In general, wakefulness was defined by high EMG activity for more than 50% of epoch duration. NREMS was defined by reduced EMG activity and increased LFP power below 4 Hz. REMS was defined by intermediate muscle tone, low LFP power over 4 Hz, and high LFP power between 6–9 Hz. A hidden Markov model is integrated into the SPINDLE scoring process to help to define the dynamics of vigilance states and suppress physiologically implausible sleep transitions. Further, an additional convolutional neural network was applied to mark unclear stages and technical artifacts so that they could be excluded from analysis. SPINDLE vigilance state classifications are exported as .csv files. The SPINDLE scoring algorithm was validated against manually scored datasets: three independent laboratories achieved average agreement rates between manual and SPINDLE state classification of 93–99% [15]. We additionally validated the algorithm in our own laboratory, where total agreement between manual and SPINDLE state classification for three 24-h mouse polysomnographic recordings was 95%.

EEG spectral data (.edfs) and vigilance state classifications (from .csv files) were processed together with MATLAB as previously described [2]. EEG spectral data were separated into the following bands: delta (1–4 Hz), theta (5–8 Hz), alpha (9–12 Hz), beta (15–35 Hz), and gamma (35–120 Hz). The gamma range was further subdivided into low gamma (35–60 Hz), gamma (60–90 Hz), and high gamma (90–120 Hz). Wakefulness was subdivided into quiet wakefulness (QW) or active wakefulness (AW) using EMG peak-to-peak amplitude of all wake epochs across the entire recording. QW was defined as the 33rd percentile or less and AW as the 66th percentile or higher of all wake EMG peak-to-peak amplitude values [16].

### 2.5. Statistical Analysis

Statistical analysis was performed with STATISTICA software (version 12.0, StatSoft, Tulsa, Oklahoma). Differences between means of sleep timings or EEG spectra were estimated by repeated-measures analysis of variance (RM ANOVA), with significance levels set to α ≤ 0.05. Partial eta squared (η^2^_p_) is reported as a measure of effect size for each significant effect in the results section. Independent variables assessed include treatment (NAC or saline injectate), sex, and time of day (12 intervals for non-cumulative sleep timing measures; 6 intervals for EEG spectral power measures). Data analysis was subjected to sigma-restricted parameterization and effective hypothesis decomposition methods by the software. Significant results were further tested by Fisher’s LSD post hoc test. While appropriate when the statistical interaction assessed is supported at α ≤ 0.05, it does not correct for multiple comparisons.

## 3. Results

### 3.1. NAC Increases Time Spent in NREM Sleep at the Cost of Wakefulness

**Figure 3:**
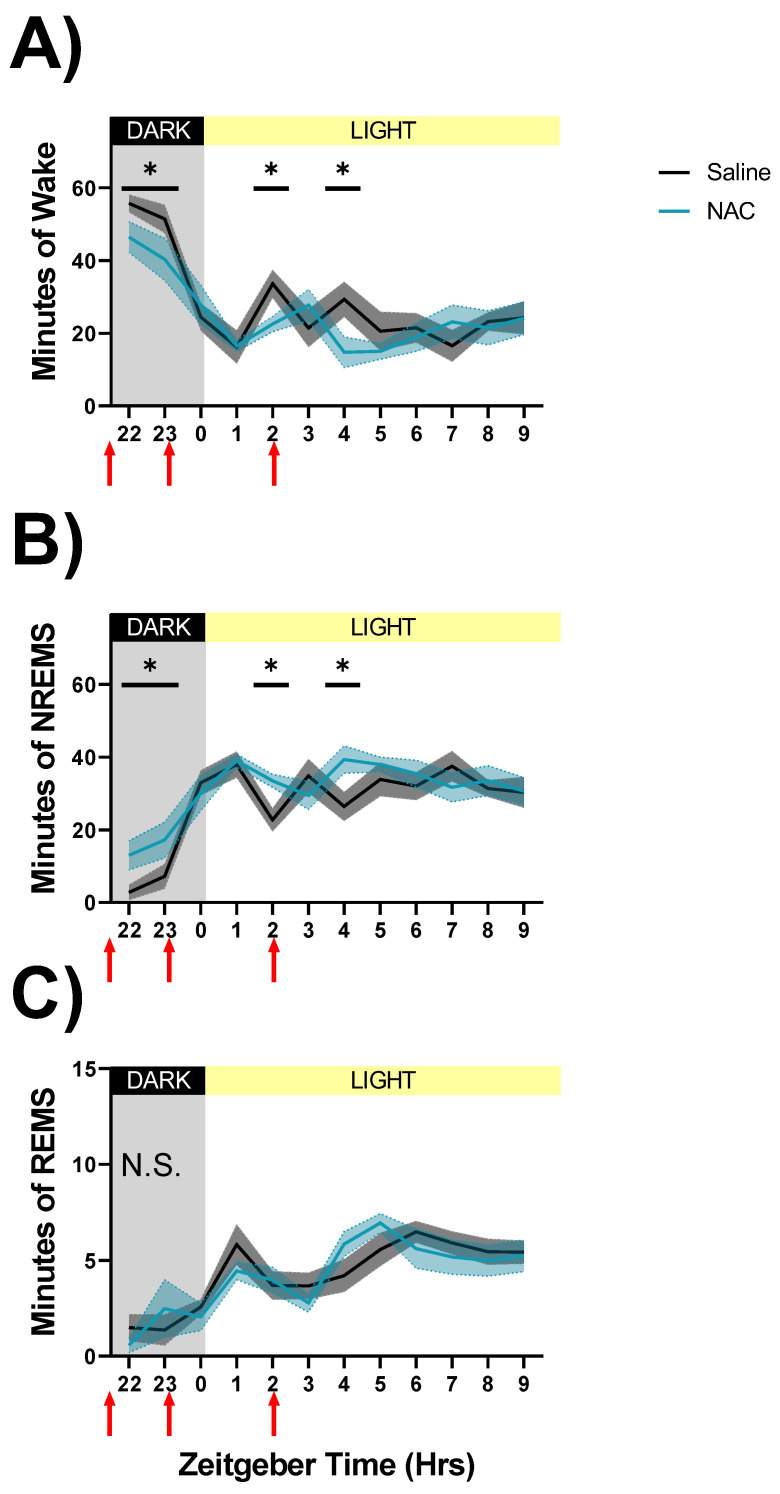
Sleep state classifications derived from SPINDLE-scoring during NAC (blue line) and saline (black line) injections on spontaneous sleep (days 30 and 31, ZT22–ZT10) recording days. Injection times are indicated by red arrows along the X-axis. Light and dark phases are denoted by the color of the background in each panel and the bar along the top of each panel. Data show the mean total number of minutes spent in each sleep state—wake (**A**), NREMS (**B**), and REMS (**C**)—during 1-h bins. Significant differences were found in wake and NREM sleep durations between NAC and saline injections during SS recordings (**A**,**B**), and indicated by asterisks where *p* < 0.05, Fisher’s LSD. Grey and light blue shaded areas signify SEM. N.S: treatment effect on REMS not significant.

We first assessed within-subjects differences in sleep architecture between EEG recordings made after NAC or saline injections during spontaneous sleep (ZT22–ZT10, days 30 & 31 in Figure 2A; data shown in Figure 3). Due to the short half-life of NAC [12,13], only the first 12 h after the first injection are displayed. RM ANOVAs indicated significant interactions of treatment x time during wakefulness (F_11,88_ = 2.51, *p* = 0.009, η^2^_p_ = 0.24, Figure 3A) and NREMS (F_11,88_ = 2.7, *p* = 0.005, η^2^_p_ = 0.25, Figure 3B). Post hoc analysis shows that NREM sleep is increased at the expense of wakefulness within the first 7 h of the injection protocol (Figure 3A,B). REMS did not show significant differences for treatment x time interaction (Figure 3C). State classifications assessed during and after sleep deprivation did not produce any significant differences between treatments (ZT0–ZT12, days 16 and 18; data not shown).

**Figure 4:**
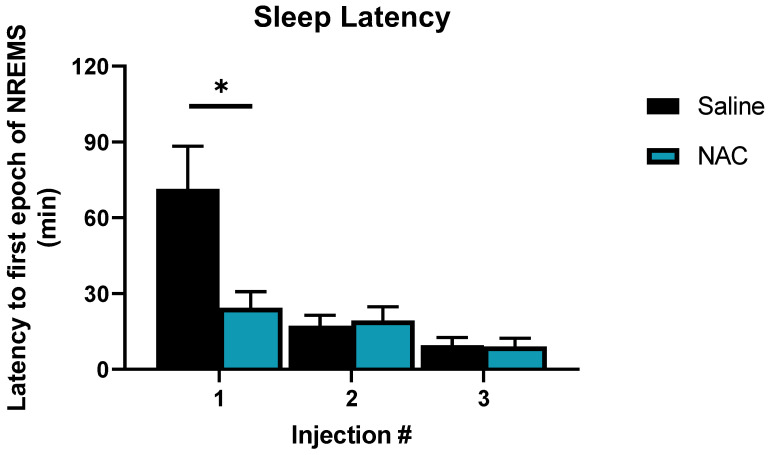
Sleep latency after NAC (blue bars) and saline (black bars) injections on SS days (days 30 and 31). Bars represent minutes elapsed after injections at ZT 22, ZT0, and ZT3, respectively, until the first epoch of NREM sleep was detected. Significant differences were found between the elapsed time after the first NAC and saline injections (at ZT 22), as indicated by the asterisks. Asterisks denote *p* < 0.05, as assessed by Fisher’s LSD post hoc test. These differences were not modulated by sex. Error bars signify SEM.

Increased time spent in NREM sleep after NAC may be explained by the reduced latency to NREM sleep onset after each of the three injections, which produced a treatment x time interaction (F_2,16_ = 9.61, *p* = 0.002, η^2^_p_ = 0.55, Figure 4). NAC treatment reduced latency to sleep onset after the first injection by 66% relative to saline (24 min for NAC vs. 71 min for saline; *p* < 0.001 post hoc: Fisher’s LSD). Latency to sleep decreased progressively after injections 2 and 3 regardless of treatment and was not affected by treatment at either of these time points. No order effect (i.e., which treatment was received on which day) was observed in latency to sleep onset.

We further probed differences in the duration of wake and NREM sleep by assessing state consolidation during the SS recordings. In the first 1-h interval beginning immediately after the first injection, wake bout duration and REMS bout duration were reduced by NAC treatment relative to saline (wake F_5,40_ = 5.01, *p* = 0.001, η^2^_p_ = 0.39; REMS F_5,40_ = 3.07, *p* = 0.019, η^2^_p_ = 0.28; data not shown). NREMS bout duration was not affected by treatment (not significant. [n.s.], data not shown). Neither the number of bouts of each state nor the number of brief awakenings was different across treatments (n.s., data not shown).

### 3.2. NAC Accelerates Changes in the Dissipation of Sleep Pressure

**Figure 5:**
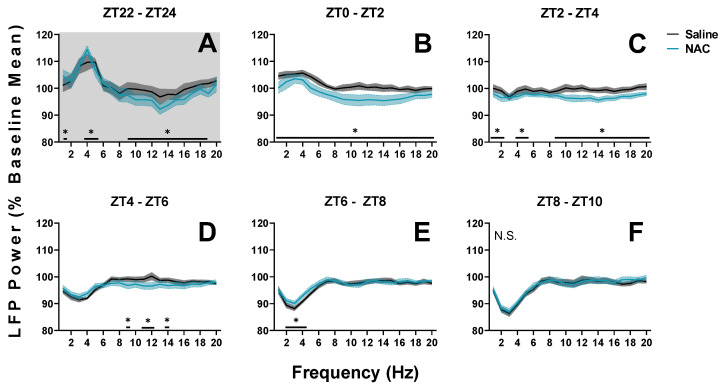
Changes in LFP spectral power during NREM sleep in mice during spontaneous sleep recordings (SS; days 30 and 31) from ZT22 to ZT10. (**A**–**F**) Panels display power in 1–20 Hz bands in sequential 2-h intervals from days when mice received either NAC (blue) or saline (black/grey) injections. Three injections were administered in this time frame at 0, 120, and 300 min; within each of the first three panels (**A**–**C**). Treatment x 2-h time interval (panel) x frequency differences between NAC and saline recordings were indicated by RM ANOVA, and individual frequency band differences were derived via post hoc assessment by Fisher’s LSD. Significance is indicated by asterisks between these groups where *p* < 0.05. These differences were not modulated by sex. Light and dark phases are denoted by the color of the background in each panel. Grey and blue shaded areas signify SEM. N.S.: No significant effect of treatment on EEG power spectra from ZT8 to ZT10.

To gain a better understanding of the increases in NREM sleep duration displayed in Figure 3B, we conducted a power analysis of low-frequency oscillations (i.e., SWA, theta, alpha, and low beta) during SS injections. When the spectral activity was separated into sleep states, significant power differences were observed in 1–20 Hz bands between NAC and saline recordings during NREM sleep (days 30 and 31 as displayed in Figure 2; RM ANOVA treatment x 2-h time interval x frequency, F_95,760_ = 1.52, *p* = 0.002, η^2^_p_ = 0.16; statistics describe all data displayed in Figure 5).

To account for time-of-day effects in response to treatments, panels in Figure 5 are separated to display low-frequency data in six two-hour time intervals from ZT 22 to ZT 10. Injections occurred 2 h prior to the start of the light phase (at ZT22; Figure 5A), at the start of the light phase (ZT 0; Figure 5B), and 3 h into the light phase (ZT3; Figure 5C). The first panel (Figure 5A) displays LFP power in low-frequency bands during the last two hours of the dark phase. This is the post-siesta period, when wakefulness generally increases, and low-frequency activity is expected to build along with sleep pressure and drowsiness. NAC recordings demonstrate increased delta power when compared to saline recordings, while alpha and low beta power are decreased (Figure 5A). Overall, this indicates that NAC accelerates the discharge of sleep drive (delta oscillations) during the dark phase.

During the light phase (Figure 5B–F), power in low-frequency bands generally decreases as increased NREM sleep duration alleviates sleep pressure and drowsiness. The first 6 h of the light phase demonstrate this effect, regardless of treatment, in Figure 5B–D. However, NAC recordings demonstrate further decreases in power across the 1–20 Hz range when compared with saline recordings. This difference suggests that sleep pressure is discharged at an accelerated rate during the early light phase when NAC is administered. Later in the light phase, a transient elevation of delta power in NAC recordings relative to saline recordings can be noted (Figure 5E), demonstrating a rebound effect after the initial acceleration of SWA discharge.

**Figure 6:**
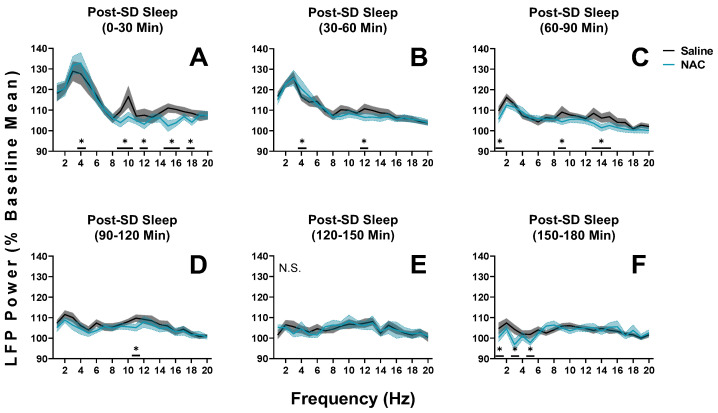
Changes in LFP spectral power during NREM sleep in mice during the hours after sleep deprivation recordings (SD; days 16 and 18; ZT6 to ZT9). (**A**–**F**) Panels display average power in 1–20 Hz bands in sequential 30-min intervals from days when mice received either NAC (blue) or saline (black/grey) injections. 30-min bins were utilized to ensure that the accelerated dynamics of SWS after SD were captured. Times displayed at the top of each panel refer to the amount of time elapsed after the sleep deprivation protocol was ended, allowing the recovery sleep opportunity to begin. No injections were administered in the timeframe of these recordings. The last injection was administered 3 h before the recordings displayed in panel A (final injection at ZT3). Treatment x 30-min time interval (panel) x frequency differences between NAC and saline recordings were indicated by RM ANOVA, and individual frequency band differences were derived via post hoc assessment by Fisher’s LSD. Significance is indicated by asterisks between these groups where *p* < 0.05. These differences were not modulated by sex. Shaded areas signify SEM.

When sleep pressure is increased after sleep deprivation, from ZT 6 to ZT 9 (post-SD; days 16 and 18 as displayed in Figure 2), the patterns described above are repeated (RM ANOVA treatment x 30-min time interval x frequency, F_95,950_ = 1.32, *p* = 0.028, η^2^_p_ = 0.12; statistics describe all data displayed in Figure 6). As expected, delta power is high after sleep deprivation, regardless of treatment, indicating increased sleep discharge. As we observed during SS (Figure 5), delta power initially increases and is discharged at an accelerated rate in NAC recordings when compared with saline recordings; at the same time, power in other low-frequency sub-bands (i.e., theta, alpha, and low-beta) is suppressed (Figure 6A–C). Eventually, delta power rebounds, decreasing during NAC recordings when compared with saline recordings (Figure 6F).

Overall, these data appear to suggest that NAC accelerates the discharge of delta power during NREMS when sleep need is elevated (i.e., during the dark phase or directly after sleep deprivation). However, in periods when sleep need has already been dissipated, animals that have been subjected to NAC injections display suppressed sleep pressure. Additionally, the initial attenuation of alpha power in NAC recordings suggests decreased cortical EEG synchronization, as alpha power is expected to increase during typical post-SD recovery sleep [17].

### 3.3. NAC Induces Sex-Specific Effects on the Accumulation of Sleep Need and Drowsiness during Enforced Wakefulness

**Figure 7:**
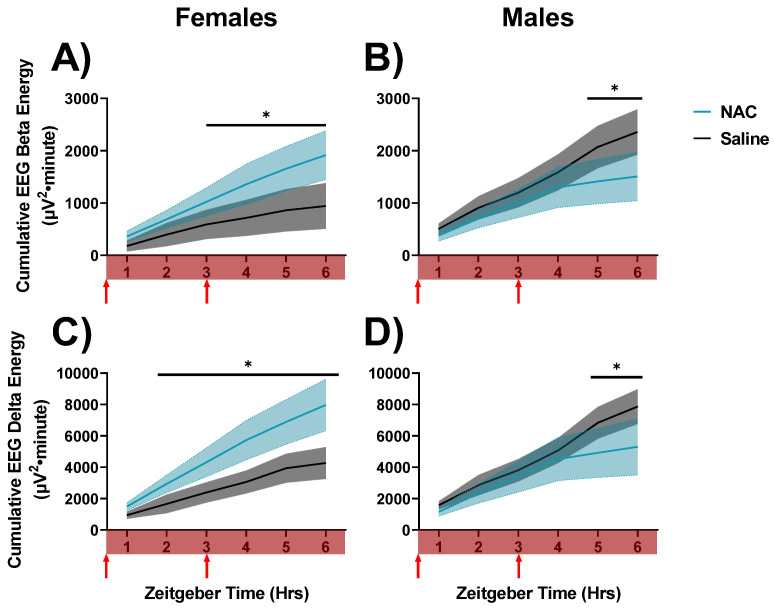
Changes in cumulated delta and beta activities during QW in SD recordings in which saline (black/grey) and NAC (blue) were administered to mice. These recordings were taken during the sleep deprivation period, as indicated by the red bar across the x-axes. Data are displayed in 1-h bins that occur from ZT0-6 on days 16 and 18 of the experimental protocol. These differences are displayed as cumulative LFP energies. Cumulative LFP energy is displayed on the left panels (**A**,**C**) for female mice and on the right (**B**,**D**) for male mice. Cumulative beta energy is displayed in the top panels (**A**,**B**), and cumulative delta energy is displayed on the bottom panels (**C**,**D**). Treatment x sex x hour differences were indicated by RM ANOVA, and differences between NAC and saline in specific intervals were derived via post hoc assessment by Fisher’s LSD. Significance is indicated by asterisks between these groups where *p* < 0.05. Injection times are indicated by red arrows. Grey and blue shaded areas signify SEM.

We next determined whether NAC impacts the dynamics of sleep needs and waking EEG activity throughout the day. To do this, we assessed cumulative LFP energies for gamma, beta, and delta oscillations during active wake (AW), quiet wake (QW), and NREM sleep during NAC and saline injections. No differences in overall LFP energies were apparent between NAC- and saline-treated animals during AW or NREMS during SD (n.s., data not shown). Cumulative gamma power during AW was also unaffected during SD (n.s., data not shown). However, NAC was found to modulate delta and beta LFP energies within QW in a sex-dependent manner during SD. In females, NAC accelerated the accumulation of delta and beta energy across QW during SD, whereas NAC suppressed the accumulation of delta and beta energy across QW during SD in males (days 16 and 18 as displayed in Figure 2; beta: F_5,40_ = 4.73, *p* = 0.002, η^2^_p_ = 0.37; delta: F_5,45_ = 6.66, *p* < 0.001, η^2^_p_ = 0.42; RM ANOVA treatment x sex x hour, assessed for post hoc differences, indicated in Figure 7). These differences indicate that NAC decreases the build-up of sleep pressure across SD in males, whereas NAC accelerates the accumulation of sleep pressure during SD in female mice. Data were also significant for treatment x sex interaction.

### 3.4. NAC Attenuates Sleep Need and Drowsiness in a Sex-Independent Manner during Quiet Wakefulness in Spontaneous Sleep

**Figure 8:**
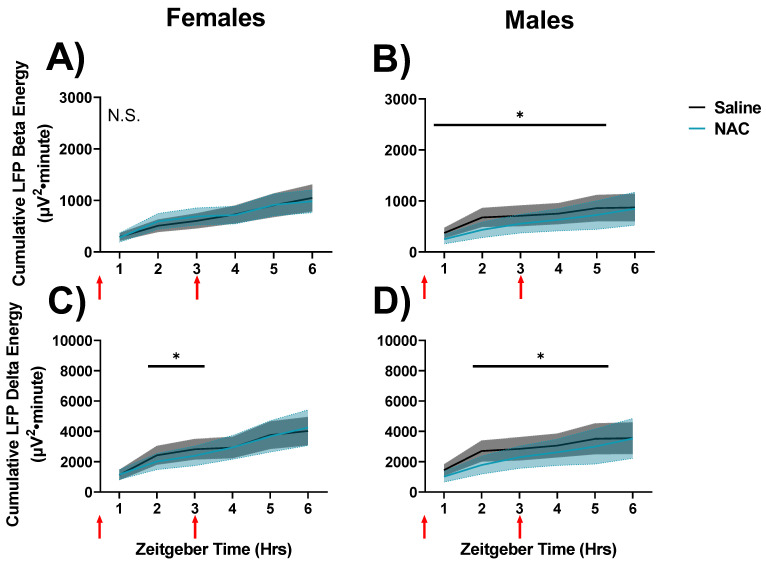
Changes in cumulated delta and beta activities during QW between SS recordings in which saline (black/grey) and NAC was administered (blue). Data are displayed in 1-h bins which occur from ZT0-6 on days 30 and 31 of the experimental protocol. Cumulative LFP energy is displayed on the left panels (**A**,**C**) for female mice and on the right (**B**,**D**) for male mice. Cumulative beta energy is displayed in the top panels (**A**,**B**), and cumulative delta energy is displayed on the bottom panels (**C**,**D**). Treatment, x time differences, were indicated by RM ANOVA, and differences between NAC and saline in specific intervals were derived via post hoc assessment by Fisher’s LSD. Significance is indicated by asterisks between these groups where *p* < 0.05. Injection times are indicated by red arrows. Grey and blue shaded areas signify SEM.

Finally, we assessed cumulative LFP energies during SS recordings (days 30 and 31). Differences were not detected between NAC- and saline-treatment recordings in cumulative delta or beta energies during NREMS in the 6-h interval after the second injection during SS recordings (n.s., not shown). Cumulative gamma energy in the active wake was also unaffected during SS recordings (n.s., not shown). However, NAC-treatments were found to attenuate cumulative beta (RM ANOVA Time x treatment interaction F_5,40_ = 2.91, *p* = 0.024, η^2^_p_ = 0.27; Figure 8A,B) and delta (RM ANOVA Time x treatment interaction F_5,40_ = 3.36, *p* = 0.014, η^2^_p_ = 0.32; Figure 8C,D) energy during QW in spontaneous sleep/wake recordings in a time-dependent but sex-independent manner. There were no effects of NAC on cumulative beta energy in female mice, but the trend was in the same direction as in male mice, which demonstrated suppression of beta energy when NAC was on board (Figure 8A,B).

Overall, delta and beta energies in NAC-treated recordings were lower than in saline-treated recordings, suggesting that NAC suppresses the accumulation of sleep need and drowsiness in QW during SS in the light phase [16].

## 4. Discussion

Here, we describe the effects of systemic redox manipulation via the glutathione precursor NAC on sleep timing and the EEG features associated with sleep homeostasis. We hypothesized that increasing the antioxidant capacity of the brain would facilitate sleep-dependent decreases in oxidative stress, decreasing the time required to dissipate sleep needs during NREMS. The effects of NAC on sleep timing and EEG parameters related to sleep homeostasis confirmed this effect, as animals exposed to NAC fell asleep faster (i.e., reduced latency to SWS) and dissipated delta power more rapidly. Overall, NAC increased the time that mice spent in NREMS at the cost of wakefulness during baseline sleep.

These effects could be a consequence of the perturbation of molecular processes that underlie sleep homeostasis, as glutathione is a regulator of sleep/wake cycles. The literature on this subject has previously described glutathione as a sleep-promoting substance and has shown that intracerebroventricular injections of oxidized glutathione (GSSG) increase time spent asleep [18,19]. It has been hypothesized that the somnogenic effects of GSSG are due to its ability to attenuate glutamatergic neurotransmission in the brain and stimulate nitric oxide synthase or general oxidative stress signaling mechanisms [19,20]. Ultimately, NAC produced the same effect, as it induced a shift in redox status that limits the ability of animals to stay awake. Additionally, NAC was observed to accelerate changes in delta power associated with sleep homeostasis. That is, when sleep need (delta power) is already increasing (post-siesta wakefulness or during SD), NAC further augments it; when sleep needs are decreasing (during sleep; lights on), NAC facilitates accelerated dissipation of delta power. Along with a general decline in EEG synchrony, this suggests that NAC changes the distribution of sleep depth by increasing initial sleep intensity without changing the timing of the accumulation of sleep needed during wakefulness.

### 4.1. N-Acetylcysteine Transiently Perturbs the Sleep Homeostat

In animals undergoing uninterrupted, spontaneous sleep/wake cycles during the light phase, delta power during NREMS declines from near peak levels at the light onset to a minimum value by the end of the light phase. Here, we have shown that NAC accelerates the rate of decline of NREMS delta power across the light phase, relative to vehicle injection.

The underlying biochemical processes which facilitate this effect of NAC are likely a direct result of increased glutathione (GSH) availability. Generally, systemic NAC is rapidly converted to glutathione via a multi-step enzymatic pathway that includes its direct conversion to L-cysteine, a rate-limiting substrate in glutathione synthesis [21]. Increased glutathione should produce additional opportunities for the production of NADPH [9], as reduced glutathione (GSH) serves as a redox substrate by undergoing oxidation to GSSG, coupled with reduction of NADP+ to NADPH, via glutathione reductase. The generation of NADPH then secondarily impacts NAD+/NADH via pathways as schematized in Figure 1. Through interconversion of NADP/NAD, the availability of GSH thus allows the cell to stabilize the NAD+/NADH ratio and NAD levels in the face of increased NAD+ consumption by enzymatic processes during wakefulness. Since fluctuations in cellular antioxidant capacity are largely due to the balance of the redox couple GSH/GSSG, this metabolic pathway is believed to be involved in mediating oxidative stress in the cell and responsible for providing the antioxidant benefits of NAC [22,23]. These data are compatible with our hypothesis that increased NAC and glutathione generally reduce sleep needs by decreasing cellular oxidative stress.

The biochemical pathway linking NAC to NAD(P):H homeostasis is especially important during prolonged wakefulness: sleep deprivation elevates brain expression of glutathione peroxidase [24] and the concentration of oxidized glutathione (GSSG) [25,26] while lowering the concentration of reduced glutathione (GSH) [27]. Accumulation of GSSG over time spent awake should decelerate glutathione peroxidase activity through product-dependent inhibition and thereby increase NADP+ concentration in the brain (as indeed occurs in sleep deprivation; [6]). Additionally, As NADPH is the primary source of reducing equivalents for glutathione, NADP+:NADPH dysregulation associated with protracted wake could endanger the efficacy of the cell’s most robust antioxidant system [28]. Ultimately, a shift in redox state limits the ability to stay awake: intracerebroventricular infusion of oxidized glutathione into the brain increases time spent asleep [19,25]. By serving as a biochemical precursor for GSH, NAC increases the pool of GSH available as a NADP+/NADPH buffering substrate.

The effects of NAC on sleep are not necessarily an exclusive consequence of its participation in glutathione-related redox and antioxidant reactions. The therapeutic potential of NAC has also been proposed to involve the modulation of glutamatergic neurotransmission in the brain [29]. The NAC metabolite L-cysteine is oxidized to L-cystine once it is taken up by tissue. Interactions with the cystine/glutamate antiporter exchange extracellular cystine for intracellular L-glutamate, especially across glial membranes [30]. This increase in extrasynaptic glutamate activates mGluR2/3 receptors, decreasing synaptic glutamate release [29]. Such decreases in excitatory activity may decrease the signaling and metabolic demands of neurons in the presence of NAC [31]. This signaling change, in addition to the conversion of NAC to glutathione, may contribute to the sleep-related changes observed here. NAC also confers other neuroprotective effects, including the stabilization of proteins and DNA by cross-linking cysteine disulfide molecules. Given that DNA damage induces sleep and that sleep has been implicated in DNA repair [32], this mechanism cannot be dismissed in considering the possible effects of NAC related to sleep. In order to use NAC to its full efficacy, further studies should explore the underlying mechanisms that specifically contribute to its sleep-related effects. Future studies should verify the extent to which acute NAC elevates cerebral glutathione in order to understand if the effects observed here are glutathione specific. Other relevant repair/oxidative stress protection mechanisms of NAC may also be relevant and worth exploring [33], including mechanisms by which NAC scavenges free radicals [34], induces neurogenesis [35], reduces mitochondrial apoptosis [36], reduces glutamatergic neurotransmission [29], and chelates metals as a form of oxidative stress protection [37].

### 4.2. Sex Differences in the Response to N-Acetylcysteine

We revealed sex differences in the impact of NAC on SD when assessing the spectral dynamics of cumulative delta and beta effects during QW (Figure 7). In females, NAC accelerated the accumulation of drowsiness and sleep needs across QW during SD, whereas in males, NAC suppressed the accumulation of drowsiness and sleep needs across QW during SD. The sex differences observed in accumulated EEG beta power during QW (i.e., drowsiness; [16]) could be the result of sex differences in antioxidant mechanisms, as males are more robustly impacted by surges of antioxidants than females. Studies assessing the capacity of human brain mitochondrial respiration have shown that mitochondria derived from males create two-fold more reactive oxygen species (ROS) than those of females [38]. An influx of glutathione via NAC would consequently be expected to have a greater impact in terms of alleviating oxidative stress in males. On the other hand, the same increase in glutathione may create reductive stress in females. If female mitochondria accumulate an excess of antioxidant capacity, further elevating GSH might free up excess NADPH to serve as a substrate for NADPH oxidase 2 (NOX2) in the generation of superoxide ions. Further, imbalances in NADP+/H and, subsequently, the dysregulated activity of NOX2 during sleep fragmentation create excessive superoxide production and subsequent inflammation. In support of this theory, NOX2-deficient mice are protected from cognitive decline associated with sleep fragmentation [39].

Final accumulated QW beta power at hour 6 during SD in the saline control condition was more than 2-fold higher in males (2358 mV^2^·min; Figure 7B) than in females (944 mV^2^·min; Figure 7A) according to post hoc comparisons of these two values (*p* = 0.027, Fisher’s LSD). This sex difference is effectively nullified by NAC, in the sense that the final accumulated beta power at hour 6 during SD in the NAC condition was (non-significantly) higher in females (1917 mV^2^·min; Figure 7A) than in males (1508 mV^2^·min; Figure 7B).

This pattern was replicated by QW delta power: Final accumulated QW delta power at hour 6 during SD in the saline control condition was higher in males (7873 mV^2^·min; Figure 7D) than in females (4268 mV^2^·min; Figure 7C; *p* = 0.049, Fisher’s LSD). This sex difference is effectively nullified by NAC, as the final accumulated delta power at hour 6 during SD in the NAC condition was (non-significantly) higher in females (7972 mV^2^·min; Figure 7C) than in males (5305 mV^2^·min; Figure 7D). The nullification of these sex differences by NAC implicates underlying sex differences in wake-dependent redox dynamics. Interestingly, during QW during SS in the light phase, similar sex differences were not observed, as NAC slowed the accumulation of delta oscillations in both males and females (Figure 8). This suggests that these sex differences may be masked under conditions where oxidative stress is not exacerbated (such as during SD).

### 4.3. Limitations of C57BL/6J as an Experimental Model

C57BL/6J mice are known to have mutations in the nicotinamide nucleotide transhydrogenase (NNT) gene, which results in mitochondrial redox abnormalities. Although this mutation spontaneously arose nearly four decades ago, it was only discovered in 2005, and C57BL/6J mice are still commonly used in laboratory experiments [40]. NNT is an enzyme that is localized to the inner mitochondrial membrane. Its main role is to reduce NADP+ to NADPH at the expense of NADH oxidation and H+ re-entry to the mitochondrial matrix. In doing so, it provides a major pathway for NADP+/H and NAD+/H interconversion within the cell. In the absence of NNT, redox-related imbalances abound with relatively minimal impact on overall health. Redox challenges of NNT-deficient mice that are pertinent to our manipulations include higher rates of hydrogen peroxide (i.e., ROS) release and poorer ability to metabolize peroxide; spontaneous NADPH oxidation; and increased ratio of oxidized glutathione to reduced glutathione (GSSG:GSH). Overall, this results in increased oxidative stress and decreased glutathione-based antioxidant capacity in these mice [40].

NAC has demonstrated neuroprotective effects in C57BL/6J mice in the literature [41,42,43]. The pathways which utilize nicotinamide-based substrates in the body are rich with redundancies—about 200 enzymes in total can process nicotinamide-based substrates; these redundancies may explain why NNT deficiency does not produce major adverse health effects in mice [40]. A comprehensive phenotypic comparison of C57BL6/J mice against C57BL6/N mice, in which NNT is repaired, additionally demonstrates that NNT-deficient C57BL6/J mice perform better in certain neurobehavioral assays of cognitive function [44]. As the NNT mutation decreases the effectiveness of NADPH-related antioxidant pathways, the metabolic impairments produced by the NNT mutation in C57BL6/J mice might be able to be considered a constitutive model of oxidative stress. Demonstrating the efficacy of NAC in this model allows for a specific understanding of the utility of such treatments in the context of increased oxidative stress in many neurological diseases. Future studies should assess whether the effects of NAC on EEG and sleep differ between C57BL/6J and other strains with intact nicotinamide pathways (such as C57BL/6N mice).

### 4.4. Translational Relevance of the Findings

The effects of NAC on sleep are likely to have translational relevance. NAC has been applied in human subject trials in neurologic and psychiatric conditions in which oxidative stress is believed to contribute to pathogenesis [45,46,47]. A prominent line of reasoning about the pathogenesis of schizophrenia, for instance, is that the schizophrenic brain is constitutively vulnerable to oxidative insults and that typically benign oxidative challenges can cause changes to the schizophrenic brain that manifest as symptoms [45]. This line of reasoning led to the hypothesis that pharmaceutical and/or nutraceutical manipulations that increase the availability of antioxidant substrates in the brain could be of therapeutic benefit. Despite the clear rationale for the use of antioxidants such as NAC as adjunct therapies in schizophrenia, clinical trials involving antioxidant administration to schizophrenic patients have led to equivocal results, with only a subset of trials finding statistically and clinically significant benefits [48,49,50]. Reasons for discrepancies in the outcome of clinical trials are not known, but results of our studies suggest that both time of day (i.e., the timing of antioxidant administration relative to sleep timing) and sex ought to be considered in the application of NAC.

Time of day significantly impacts oxidation-reduction reactions in the brain [51,52,53], which may influence the efficacy of antioxidant therapeutics in the treatment of neurologic and psychiatric conditions. Sleep is associated with dramatic shifts in the oxidative status of biochemical substrates in the brain and supports mechanisms that reduce accumulated oxidative stress that builds during waking activities [54,55,56]. Disruption of sleep and its associated neuronal network activities is a hallmark of schizophrenia [57,58]. For example, sleep acutely attenuates abnormalities in sensory gating in schizophrenia [59] and similar sensory gating deficits that emerge in non-schizophrenics voluntarily undergoing habitual sleep restriction [60]. Our experiment demonstrates the somnogenic qualities of NAC, which is replicated in experimental infusions of GSSG [18,19].

**Figure 9:**
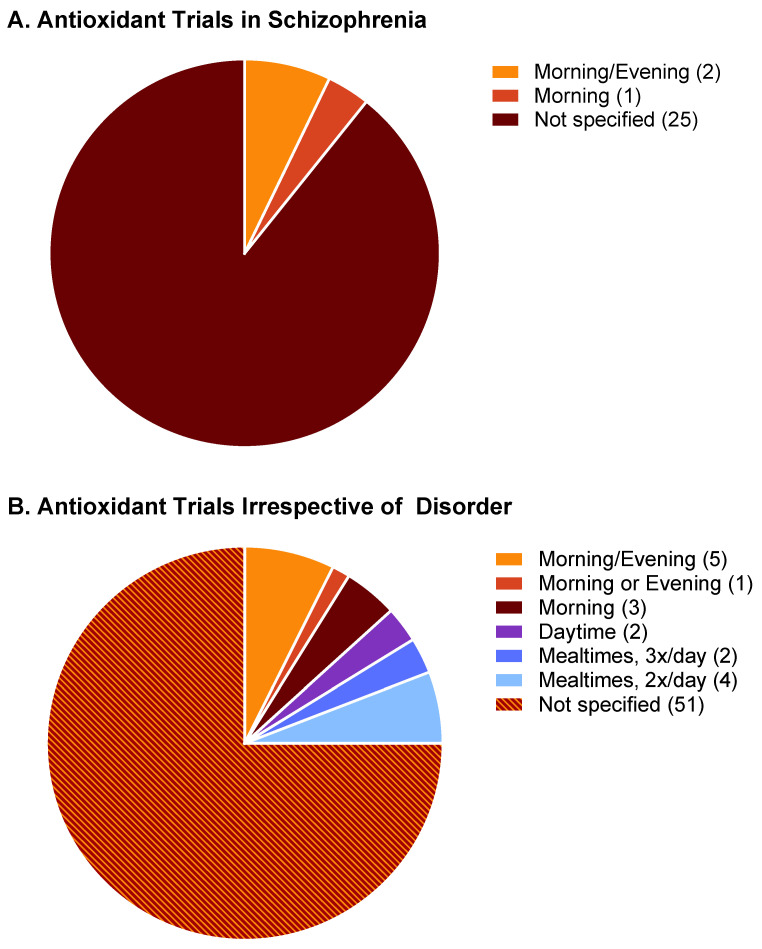
Timing of administration of NAC and related antioxidants in clinical trials. (**A**) Data from 32 clinical trials involving schizophrenia. (**B**) Data from 93 clinical trials reviewed without regard to the disorder targeted. Numbers in parentheses indicate the number of trials included in that category.

Because the oxidative insult of sleep disruption exacerbates the symptoms of schizophrenia, manipulations that increase the availability of antioxidant substrates may benefit from being timed relative to the sleep/wake cycle. Specifically, we hypothesize that for antioxidants taken for the purpose of targeting and protecting the brain (as is the case of NAC for schizophrenia), maximal therapeutic efficacy will occur when the antioxidant is administered at a time and in a manner that delivers the antioxidant to the brain when sleep need is highest, at sleep onset. Conversely, therapeutics administered without regard for sleep-dependent antioxidant processes in the brain may not deliver positive results. We attempted a meta-analysis of studies to test this hypothesis. Pubmed searches were conducted for the following term sets, with filtering for clinical trials: “schizophrenia nicotinamide,” “schizophrenia niacin”, “schizophrenia acetylcysteine”. Collectively, the three searches yielded access to 28 published studies, the PMIDs of which are included as supplementary materials with this manuscript (Supplementary Table S1). Trials involved the administration of five agents: N-acetylcysteine (*n* = 17); niacin or niacinamide (*n* = 4); nicotinic acid (*n* = 2); nicotinamide adenine dinucleotide (*n* = 3); nicotinamide (*n* = 2). Of the 28 trials accessed, only three specified the time of day at which the agent was administered (Figure 9A). Two trials involved one morning dosing and one evening dosing, and one trial involved a morning dose exclusively. No trials engaged in a systematic comparison of exclusive morning vs. exclusive evening dosing; no trials employed exclusively evening dosing. Thus, 89% of clinical studies examining the efficacy of N-acetylcysteine and related antioxidants in schizophrenia do not report the time of day of N-acetylcysteine administration, and no studies appear to have systematically varied time of day as part of the design. Of those 28 studies, only 20 studies (71%) assessed study outcomes in both sexes, and only 7 (25%) reported statistical analysis related to sex differences (Supplementary Table S1), with many studies claiming an inability to properly assess sex differences, given small sample sizes.

A more general Pubmed review of clinical trials examining the efficacy of nicotinamide-based antioxidants without regard to the target disease state indicated that 76% do not report the time of day of administration (Supplementary Table S2). Pubmed searches were conducted for the following term sets, with filtering for clinical trials: “nicotinamide supplement”, “nicotinamide supplementation”, “dietary nicotinamide”, “n-acetylcysteine supplement”, “dietary n-acetylcysteine”, “dietary n-acetyl cysteine”, “n-acetyl cysteine supplement”, “nicotinamide riboside”, or “nicotinamide mononucleotide”. Resulting accessible trials involved the administration of 7 agents: N-acetylcysteine (*n* = 37); niacin or niacinamide (*n* = 4); nicotinamide (*n* = 7); nicotinamide adenine dinucleotide (NAD; *n* = 4); nicotinamide mononucleotide (*n* = 4); nicotinamide riboside (*n* = 11); or nicotinic acid (*n* = 1). The vast majority of studies did not report the time of day of antioxidant administration (51 of 68 studies; Supplementary Table S2). Five studies involved only daytime or morning administration; no trials have employed exclusively evening dosing. Six of the studies incorporated morning and evening administration; five of these entailed morning administration in addition to evening administration in the same subject group. A single study (PMID 35215405) systematically compared the effects of nicotinamide mononucleotide (NMN) supplementation in the morning vs. evening on self-reported, subjective sleep quality and drowsiness. Subjects were instructed to take supplements either in the AM (between wake-up time and 12:00) or PM (between 18:00 and bedtime). Those in the PM NMN group had reduced daytime drowsiness ratings relative to PM placebo controls, whereas those in the AM NMN group did not differ from AM placebo controls. This study, which appears to be unique to the literature in that it systematically assessed time-of-day effects on antioxidant efficacy, is suggestive of a time-of-day-dependent antioxidant response relevant to sleep and sleepiness (Figure 9B). Future studies should further address this possibility and, at a minimum, specify in reports the time of day at which antioxidants were administered.

Of the 68 studies included in this general review of nicotinamide-based antioxidants, only 39 (57%) assessed study outcomes in both sexes, and only 9 (13%) reported statistical analysis related to sex differences (Supplementary Table S2). Out of the studies which reported statistical analysis of sex differences, four studies (6%) found differences between sexes. Two of these studies found that nicotinamide treatments were more effective in males (PMIDs 8951265, 7660324), whereas two found more striking results in females (PMIDs 34238308, 32320006), with several studies mentioning that larger study sizes would be necessary to properly assess the difference in sex. These equivocal results comport with our understanding of sex differences, as observed in Figure 7 and Figure 8, which suggest that sex differences may reveal themselves differentially based on a subject’s prior accumulation of tissue-level oxidative stress (which may be roughly assessed by time of day).

Disorders where NAC or other nicotinamide-based treatments are commonly used as adjunctive therapies are often conditions in which sleep is taxed and tissue-level oxidative stress is high. Given these results, we suggest that future clinical studies assess both sexes, as conditions in which oxidative stress is exacerbated will likely reveal sex differences (as suggested by PMID 32320006). Consideration of both time of day and sex together could potentially improve the replicability of study outcomes and the efficacy of antioxidant-based treatments in clinical trials.

## 5. Conclusions

The studies described here demonstrate that N-acetylcysteine modulates the sleep-wake cycle in mice: reducing the latency to onset of and increasing the amount of time spent in NREMS. The sleep-promoting effect of N-acetylcysteine may contribute to its therapeutic potential in schizophrenia and other neuropsychiatric conditions, provided that it is administered at bedtime. N-acetylcysteine may modulate the electroencephalogram differently in male and female subjects. It is, therefore, recommended that future clinical trials involving N-acetylcysteine incorporate bedtime administration into the design and measure the effects of treatments in both male and female subjects.

## Data Availability

The data presented in this study are available on request from the corresponding author.

## Supplementary Materials

The following supporting information can be downloaded at: https://www.mdpi.com/article/10.3390/antiox12051124/s1, Table S1: Antioxidant Trials in Schizophrenia; Table S2: Antioxidant Trials Irrespective of Disorder.
